# Association Between Salt Intake Habits and Vasomotor Symptoms According to Adiposity Among Middle-Aged Korean Women: A Longitudinal Cohort Study

**DOI:** 10.3390/nu18213618

**Published:** 2026-10-31

**Authors:** Yoonyoung Jang, Soo Min Kim, Sujeong Shin, Jeonggyu Kang, Yoosun Cho, Yoosoo Chang, Seungho Ryu

**Affiliations:** 1 Center for Cohort Studies, Kangbuk Samsung Hospital, Sungkyunkwan University School of Medicine, Seoul 04514, Republic of Korea; jyy0227@naver.com (Y.J.);; 2 Institute of Medical Research, Sungkyunkwan University School of Medicine, Suwon 16419, Republic of Korea; 3 Office of Policy Research, National Food Safety Information Service, Seoul 04514, Republic of Korea; smkim.stat@gmail.com; 4 Department of Family Medicine, Kangbuk Samsung Hospital, Sungkyunkwan University School of Medicine, Seoul 04514, Republic of Korea; 5 Asan Medical Center, University of Ulsan College of Medicine, Seoul 04514, Republic of Korea; 6 Department of Occupational and Environmental Medicine, Kangbuk Samsung Hospital, Sungkyunkwan University School of Medicine, Seoul 04514, Republic of Korea; 7 Department of Clinical Research Design & Evaluation, Samsung Advanced Institute for Health Sciences & Technology, Sungkyunkwan University, Seoul 04514, Republic of Korea

**Keywords:** salt intake habits, vasomotor symptoms, hot flashes, night sweats, adiposity

## Abstract

**Objectives:** Whether habitual salt consumption is related to vasomotor symptoms (VMS) during the menopausal transition has received little attention. We investigated the relationship of salt-related eating behaviors with bothersome VMS over time and explored whether body composition modified this relationship. **Methods:** We analyzed repeated observations from 3664 Korean women who entered the cohort between 2014 and 2018 and were followed until July 2024. A three-item questionnaire was used to generate a salt intake habit score, which was divided into tertiles. Bothersome VMS across repeated visits were analyzed using logistic mixed-effects models with inverse probability weighting, and effect modification by adiposity was explored. **Results:** At enrollment, mean age was 45.6 years; among women with repeated assessments, the median observation period was 5.6 years. Relative to the lowest salt intake tertile, adjusted odds ratios (95% CI) for bothersome VMS were 1.48 (1.22–1.80) in the middle tertile and 1.51 (1.20–1.89) in the highest tertile. The association appeared stronger among women with percentage body fat ≥35% (*p* for interaction = 0.032), although the interaction did not reach the Bonferroni-corrected threshold (*p* < 0.0167). **Conclusions:** Saltier eating habits were related to a greater likelihood of bothersome VMS during follow-up. These findings should be considered hypothesis-generating, and whether reducing salt consumption also decreases VMS warrants testing in intervention studies.

## 1. Introduction

Vasomotor symptoms (VMS) affect 50–75% of women as ovarian function declines and menstrual cycles become irregular [1]. Typical manifestations include hot flushes, night sweats, and episodes of excessive sweating [2]. Although traditionally regarded primarily as menopausal discomfort, accumulating evidence links VMS with subsequent health outcomes extending beyond symptom burden itself [3]. Epidemiologic studies have reported associations with cardiovascular risk and events [4], as well as with skeletal, metabolic, psychological, and cognitive outcomes [5,6,7,8,9]. These observations have shifted attention toward factors that might be modified before or during the menopausal transition to reduce the occurrence or persistence of VMS.

Excess sodium intake can adversely affect cardiovascular physiology by altering blood pressure regulation, arterial stiffness, and endothelial function [10,11,12]. The pathophysiological mechanisms underlying VMS involve vascular dysfunction and autonomic dysregulation [13,14]—processes that are also directly influenced by excessive sodium intake [15]. Given this mechanistic overlap between VMS and cardiovascular risk, habitual salty eating patterns may plausibly contribute to VMS occurrence and severity. Body composition may further influence this relationship, as greater overall and abdominal adiposity have been associated with VMS, possibly through the thermoregulatory effects of body fat [16,17]. Nevertheless, research directly examining the association between salt intake habits and VMS—and whether this association differs according to body composition—remains scarce.

We therefore examined whether salt-related eating behavior was associated with VMS over follow-up and whether this relationship varied according to adiposity. VMS assessed repeatedly during follow-up were analyzed, with percentage body fat, waist circumference, and BMI considered as potential modifiers.

## 2. Materials and Methods

### 2.1. Study Population

The analytic cohort originated from the Korean Longitudinal Study of Midlife Women, conducted at the Kangbuk Samsung Hospital Total Healthcare Center [18,19,20]. Recruitment took place from 2014 through 2018 among women aged 42–52 years, and subsequent assessments continued until July 2024. At study entry, baseline reproductive status was limited to premenopause or early transition. Follow-up visits were scheduled annually or biennially and included repeated questionnaire-based assessments [18,19,20]. Women were eligible if they had not undergone hysterectomy or oophorectomy, had not received hormone replacement therapy, and had not experienced an episode of amenorrhea lasting 60 days or longer at baseline. Women with kidney failure, malignancy, hypothyroidism, or hyperthyroidism were also excluded because these conditions could interfere with menstrual cyclicity [21]. All participants provided written consent after receiving information about the study.

**Figure 1:**
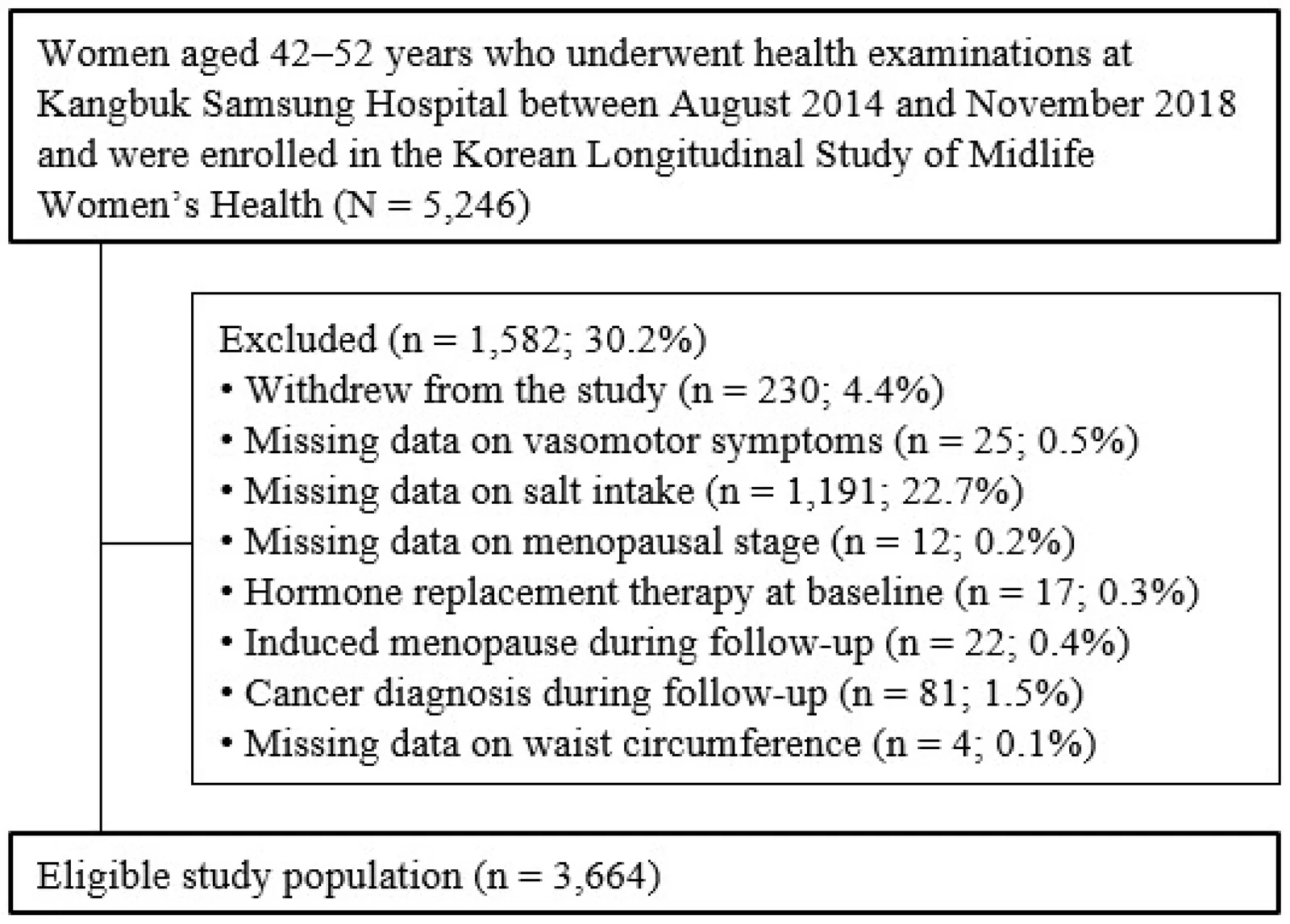
Flowchart of participant selection.

Figure 1 shows the participant selection process. Of the 5246 women initially enrolled, 1582 were excluded: 230 withdrew from the study; 25 had missing data on VMS, 1191 on salt intake habits, 12 on menopausal stage, and 4 on waist circumference; 17 used hormone replacement therapy at baseline; 22 experienced induced menopause during follow-up; and 81 were diagnosed with cancer during follow-up. Thus, 3664 participants constituted the study population. Additionally, for the sensitivity analysis of incident bothersome VMS, 2061 participants with only a baseline measurement and 291 with bothersome VMS at baseline were further excluded, resulting in 1312 participants.

The study protocol received approval from the Institutional Review Board of Kangbuk Samsung Hospital (IRB No. KBSMC 2026-03-065). All study procedures were performed under the approved protocol and relevant institutional requirements.

### 2.2. Measurement

Sociodemographic, reproductive, behavioral, and medical information was obtained from standardized participant questionnaires. Smoking was categorized as never versus current/former smoking, with the latter defined by a lifetime consumption of at least 100 cigarettes [22]. Alcohol intake was classified into two groups based on average daily consumption: <10 g/day or ≥10 g/day [23]. Physical activity was evaluated with the Korean short-form International Physical Activity Questionnaire (IPAQ) and grouped into health-enhancing physical activity (HEPA), moderately active, or inactive categories according to established IPAQ criteria [24,25]. Body mass index (BMI) was classified as <23.0, 23.0–24.9, or ≥25.0 kg/m^2^ [26]. For analyses examining modification by adiposity, waist circumference was divided at 80 cm [27], and percentage body fat at 35% [28].

Age at menarche was grouped as <12, 12–13, 14–16, or ≥17 years. Participants were classified as nulliparous or parous. Marital status was grouped as unmarried, married/cohabiting, or divorced/separated/widowed, and educational attainment as high school or less versus college or higher. Hypertension, diabetes, and hyperlipidemia were coded according to whether a prior history was present.

### 2.3. Salt Intake Habits

Salt-related eating habits were assessed with a three-item questionnaire developed for Korean adults [29] and previously applied in a large Korean health screening population [30]. In the development study, 15 candidate items, six on salt usage behaviors and nine on the consumption of high-salt foods, were compared with sodium excretion in a single 24 h urine collection from 189 normotensive adults [29]; the three behavior items that showed significant Spearman’s partial correlations with urinary sodium after adjustment for age, sex, and BMI (r = 0.17–0.19) were retained [29]. Although each retained item showed only a weak correlation, the summed score showed a modest partial correlation with 24 h urinary sodium excretion (r = 0.26, *p* < 0.001) [29].

The first item, “What do you think of your salt-eating habits?”, had five responses scored in ascending order of saltiness: very bland (0 points), slightly bland (3), average (6), slightly salty (9), and very salty (12) [30]. The second item, “Do you add salt or soy sauce on cooked dishes?”, was scored as never (0), seldom (4), frequently (8), or always (12) [30]. Soy sauce is a major sodium-containing condiment in Korean meals; in samples collected nationwide from households, foodservice establishments, and restaurants, its mean sodium content was 5827 mg per 100 g [31]. The third item, “Do you eat pan-fried or deep-fried food with soy sauce?”, was scored as never (0), sometimes (6), or always (12) [30]. In an independent study of Korean women, responses to this item were correlated with sodium intake estimated by 24 h dietary recall among middle-aged women (mean age, 48 years; Spearman’s r = 0.44), but not among younger female college students (mean age, 21 years) [32]. This finding supports the applicability of the item to a middle-aged population similar to ours, while not replacing direct validation in the present cohort.

The first item thus captures a general tendency toward salty eating, whereas the second and third items capture specific practices of adding salt or soy sauce to cooked food. Although these items may partly overlap, the summed score showed a stronger correlation with urinary sodium than any single item in the development study [29], suggesting that they capture partly distinct aspects of salt-related eating behavior. The three item scores were summed to give a total score of 0 to 36, with higher values indicating saltier eating behavior. In a large Korean health screening population from the parent cohort of the present study, a higher salt intake score was associated with higher sodium intake estimated by a food frequency questionnaire [30]. Because urinary sodium was not measured in the present cohort, the score was therefore regarded as an indicator of habitual salt-related eating behavior rather than a quantitative estimate of sodium intake. As no validated clinical threshold has been defined for this score [29], categories were based on tertiles of the baseline distribution: ≤10, 11–16, and ≥17. The same thresholds were applied to repeated measurements during follow-up.

### 2.4. Vasomotor Symptoms (VMS)

VMS were assessed using the vasomotor domain of the Menopause-Specific Quality of Life (MENQOL) questionnaire [33,34], which comprises three items: hot flushes, night sweats, and sweating. Each item is rated as 1 (not experienced), 2 (experienced but not bothersome), or 3–8 (experienced and bothersome, with higher scores indicating greater bother), and the domain score was calculated as the mean of the three items. The primary outcome was bothersome VMS, defined as a domain score > 2, indicating that symptoms were, on average, bothersome. A score ≤ 2 indicates either no symptoms or symptoms that were present but not bothersome. The cutoff of > 2 was chosen because more severe VMS have been associated with a substantial burden on quality of life [35]. At baseline, the domain score was right-skewed, and 20% of participants had a score >2 (Figure S2). The domain score was also analyzed as a continuous outcome in sensitivity analyses.

### 2.5. Definition of Menopausal Stages

Menopausal status was updated at each visit using STRAW+10 definitions [36]. Premenopause was assigned when no transition criterion was present. Early transition required at least two episodes of menstrual-cycle length variation of 7 days or more within the previous year. Late transition was defined by an interval of ≥60 days without menses during the preceding year, and postmenopause by amenorrhea lasting more than 12 months.

### 2.6. Covariate Selection

A directed acyclic graph (DAG) constructed from prior evidence on the relationships among salt intake, adiposity, VMS, and related factors was used to identify covariates (Figure S1). Age, educational attainment, marital status, smoking status, and physical activity were considered potential confounders, as these factors have been associated with both dietary intake [37,38] and VMS [39,40]. Menopausal stage, age at menarche, and parity were additionally included as covariates because they are previously reported risk factors for VMS [40,41,42]. Hypertension and BMI were included as covariates in the main analysis. Salt intake has been associated with higher blood pressure [43] and greater adiposity [44], both of which have been associated with VMS [39,45]. Because a diagnosis of hypertension may itself prompt salt reduction, the main model was refitted after excluding participants with hypertension at any study visit as a sensitivity analysis. Obesity has been associated with greater salt sensitivity, possibly through insulin resistance, inflammation, renin–angiotensin–aldosterone system (RAAS) activation, sympathetic activity, and vascular dysfunction [46,47]; therefore, percentage body fat, waist circumference, and BMI were each examined as effect modifiers in separate models.

### 2.7. Statistical Analysis

Baseline variables were summarized using mean (standard deviation) for continuous measures and number (%) for categorical measures.

To account for potential selection bias, inverse probability weights were applied in all analyses. The weights were calculated as the product of two components: the inverse of the estimated probability of inclusion in the baseline analytic sample and the inverse of the estimated probability of having repeated measurements among the included participants. Each probability was estimated using logistic regression including the year of the baseline examination, age, menopausal stage, smoking status, alcohol consumption, physical activity, BMI, age at menarche, parity, marital status, and educational attainment. Baseline characteristics were compared between included and excluded participants and between participants with and without repeated measurements using the t-test for continuous variables and the chi-square test for categorical variables. Because *p*-values are sensitive to sample size, standardized mean differences (SMDs) were also calculated, with an absolute SMD > 0.1 considered indicative of a meaningful imbalance [48].

Associations between salt intake habits and bothersome VMS across repeated visits were estimated using mixed-effects logistic regression with a participant-level random intercept to account for within-person correlation. Salt intake habit tertiles and covariates were modeled as time-varying, with the exception of age at menarche, which was fixed at baseline. Model 1 included age and menopausal stage. Model 2 additionally included smoking status, parity, marital status, educational attainment, physical activity, BMI, hypertension, and age at menarche.

To explore whether the association varied by adiposity, percentage body fat (<35% vs. ≥35%), waist circumference (<80 vs. ≥80 cm), and BMI (<23 vs. ≥23 kg/m^2^) were examined in separate models, each including a product term between the salt intake habit tertile and the dichotomized body composition measure. The interaction was tested with a Wald test. To account for multiple comparisons, Bonferroni-corrected significance thresholds were applied: *p* < 0.0167 (0.05/3) for interaction across the three body composition measures and *p* < 0.0083 (0.05/6) for the six within-stratum comparisons of tertile 3 versus tertile 2. Stratum-specific odds ratios (ORs), as well as ORs contrasting tertile 3 with tertile 2 within each stratum, were derived from the same models. Predicted prevalences of bothersome VMS with 95% confidence intervals (CIs) were obtained from these models and displayed graphically. The analysis for BMI was repeated using a cut-off of 25 kg/m^2^.

Sensitivity and additional analyses were performed to assess the robustness of the findings. First, the MENQOL vasomotor domain score was analyzed as a continuous outcome using linear mixed-effects models. Second, participants with hypertension at any study visit were excluded. Third, alcohol consumption was additionally adjusted for. Fourth, salt intake habits were lagged by one study visit so that exposure assessment preceded VMS measurement. The second to fourth analyses were performed for both the binary and continuous outcomes, using the same covariates, random intercepts, and inverse probability weights as the main model. Finally, among women without bothersome VMS at the first visit, cumulative incidence of bothersome VMS by baseline salt intake habit tertile was estimated using the Kaplan–Meier method and compared using the log-rank test, and incident bothersome VMS was analyzed using Cox proportional hazards models, with salt intake habits and covariates treated either as baseline measures or as time-varying variables. In the time-dependent models, values measured at each visit were applied to the subsequent risk interval and used to predict bothersome VMS identified at the next assessment. Inverse probability weights were applied to account for selection into the Cox analytic sample.

Results are presented as ORs, β coefficients, and HRs with 95% CIs. All analyses were conducted in Stata 18.0 (StataCorp, College Station, TX, USA) and R version 4.4.2 (R Foundation for Statistical Computing, Vienna, Austria). Statistical significance was defined using a two-sided α of 0.05, except for the interaction analyses, for which Bonferroni correction was applied.

## 3. Results

Table 1 describes the study population at enrollment. Participants had a mean age of 45.6 years (SD, 3.1). Among women contributing repeated observations, follow-up lasted a median of 5.6 years (IQR, 3.8–7.6), with a median interval of 2.0 years (IQR, 1.0–4.0) between assessments. For most baseline characteristics, absolute SMDs were below 0.1, both between included and excluded participants (Table S1) and between those with and without repeated measurements (Table S2). Excluded participants were younger, and those with only a baseline measurement were more often postmenopausal.

Higher salt intake habit scores were associated with higher odds of bothersome VMS (Table 2). Compared with tertile 1, the ORs in Model 2 were 1.48 (95% CI 1.22–1.80) for tertile 2 and 1.51 (95% CI 1.20–1.89) for tertile 3, with similar estimates in Model 1. The odds of bothersome VMS increased with time-varying age (OR per 1-year increase, 1.14; 95% CI 1.11–1.18) and with menopausal stage; compared with premenopause, the ORs were 1.47 (95% CI 1.16–1.87) for the early transition, 3.26 (95% CI 2.48–4.28) for the late transition, and 4.10 (95% CI 2.99–5.62) for postmenopause. Among the other covariates, BMI (23.0–24.9 kg/m^2^: OR 1.46, 95% CI 1.17–1.82; ≥25.0 kg/m^2^: OR 2.49, 95% CI 1.95–3.19; reference <23.0 kg/m^2^) and hypertension (OR 1.69, 95% CI 1.20–2.37) were also associated with higher odds of bothersome VMS (Table S3).

In exploratory analyses of whether the association differed by body composition, the *p* for interaction was 0.032 for percentage body fat, 0.085 for waist circumference, and 0.289 for BMI; none met the Bonferroni-corrected threshold (*p* < 0.0167) (Table 3). Among women with percentage body fat ≥35%, the OR for tertile 3 was 2.24 (95% CI 1.46–3.46), and the OR contrasting tertile 3 with tertile 2 was 1.55 (95% CI 1.04–2.32; *p* = 0.030), which did not reach the corrected threshold (*p* < 0.0083). None of the other within-stratum comparisons of tertile 3 versus tertile 2 were statistically significant, even before correction (*p* = 0.218). In terms of predicted prevalence, bothersome VMS was more prevalent in the higher adiposity strata and tended to increase across salt intake habit tertiles in these strata, whereas in the lower adiposity strata it increased from tertile 1 to tertile 2 without a further increase in tertile 3 (Figure S3). When BMI was dichotomized at 25 kg/m^2^, no interaction was observed (*p* for interaction = 0.982), and the patterns across tertiles were similar in both strata (Figure S4).

The sensitivity and additional analyses yielded results generally consistent with the main analysis. When the MENQOL vasomotor domain score was analyzed as a continuous outcome, higher salt intake habit scores were associated with higher VMS scores (tertile 2: β = 0.08, 95% CI 0.02–0.15; tertile 3: β = 0.15, 95% CI 0.07–0.23; Table S4). The associations remained after excluding participants with hypertension at any study visit (tertile 2: OR 1.52, 95% CI 1.24–1.87; tertile 3: OR 1.55, 95% CI 1.21–1.97; Table S5) and after additional adjustment for alcohol consumption (tertile 2: OR 1.47, 95% CI 1.21–1.79; tertile 3: OR 1.46, 95% CI 1.16–1.84; Table S6). With exposure lagged by one study visit, the association was observed for tertile 3 (OR 1.64, 95% CI 1.18–2.28), whereas the estimate for tertile 2 did not reach statistical significance (OR 1.26, 95% CI 0.95–1.66; Table S7). In these three analyses, the continuous VMS score was significantly higher in tertiles 2 and 3 than in tertile 1 (excluding hypertension: β = 0.09 and 0.15; alcohol adjustment: β = 0.08 and 0.14; lagged exposure: β = 0.12 and 0.23, respectively; Tables S5–S7). In the analysis of incident bothersome VMS, the cumulative incidence did not differ significantly across baseline tertiles (log-rank *p* = 0.073; Figure S5). In the time-dependent Cox model, higher salt intake habit scores tended to be associated with a higher risk of incident bothersome VMS (tertile 2: HR 1.26, 95% CI 1.02–1.54; tertile 3: HR 1.23, 95% CI 0.97–1.56; *p* for trend = 0.053; Table S8).

## 4. Discussion

Higher salt intake habit scores were related to higher odds of bothersome VMS over follow-up. After multivariable adjustment, women in tertiles 2 and 3 of the salt intake habit score had 48% and 51% higher odds of bothersome VMS, respectively, than those in tertile 1. The association remained generally consistent when the vasomotor domain score was analyzed as a continuous outcome and when salt intake habit scores were lagged by one study visit. In the analysis of incident bothersome VMS, higher salt intake habit scores tended to be associated with a higher risk. In exploratory analyses, the association appeared more pronounced among women with percentage body fat ≥35%, particularly in the highest tertile of the salt intake habit score; however, this interaction did not reach the Bonferroni-corrected significance threshold (*p* for interaction = 0.032; threshold *p* < 0.0167). No statistically significant interaction was observed for waist circumference or BMI (*p* for interaction = 0.085 and 0.289, respectively).

While previous studies have examined overall dietary patterns in relation to VMS, focusing primarily on plant-based diets and soy intake [49,50,51], our findings suggest that habitual salt-related eating behavior may also be associated with VMS. This may be particularly relevant in populations with traditionally high sodium consumption, such as Korea [52]. Notably, current major menopause guidelines emphasize hormone therapy, non-hormonal pharmacologic agents, weight management, smoking cessation, and alcohol reduction for VMS management, but do not identify sodium reduction as a specific preventive or therapeutic strategy [53]. Because salt sensitivity of blood pressure has been reported to increase in midlife and postmenopausal women [54], and sodium reduction is already recommended for cardiovascular risk reduction [43], it would be worthwhile to examine in intervention studies, including randomized controlled trials, whether reducing salt intake also reduces the frequency or severity of VMS.

Several biological mechanisms may explain the association between high salt intake and VMS. High sodium intake increases intravascular volume and blood pressure, activates the sympathetic nervous system, and may blunt the normal suppression of the renin–angiotensin–aldosterone system, particularly in salt-sensitive individuals [55]. These hemodynamic changes impair vascular relaxation and reduce nitric oxide bioavailability [56], key factors in maintaining endothelial function. These hemodynamic and vascular changes may decrease the threshold for thermoregulatory instability in the hypothalamus, leading to exaggerated vasodilatory and sudomotor responses that present as hot flushes, nocturnal hyperhidrosis, and excessive sweating [57]. Additionally, excessive sodium consumption has been correlated with low-grade systemic inflammation and unfavorable metabolic profiles [58], both of which are associated with VMS and increased cardiovascular risk [59]. Declining estrogen levels during the menopausal transition may further heighten susceptibility to sodium-related vascular and thermoregulatory disturbances by increasing salt sensitivity and altering vascular regulation [54,60].

Exploratory analyses suggested a stronger association among women with higher percentage body fat, but the interaction did not meet the Bonferroni-corrected significance threshold. Percentage body fat may capture total adiposity more directly than BMI, which does not distinguish fat from lean mass, while waist circumference cannot distinguish subcutaneous from visceral fat. Prior studies have linked total and subcutaneous, but not visceral, abdominal adiposity with hot flashes, and longitudinal data have related gains in body fat to greater VMS reporting [16,28,61,62]. Because adipose tissue conducts heat less efficiently than lean tissue, greater fat mass may impede heat loss and thereby increase susceptibility to hot flashes [16,63,64]. High salt intake may also adversely affect endothelial, metabolic, and inflammatory pathways [65,66,67], which could have greater consequences in women with excess adiposity. Interindividual susceptibility to sodium may further contribute to this heterogeneity. Although salt sensitivity has been established primarily for blood pressure, it is more common in women and is associated with obesity, insulin resistance, inflammation, and vascular dysfunction, raising the possibility that some women with excess adiposity may be more susceptible to sodium-related physiological perturbations relevant to VMS [66,67]. However, because these analyses were exploratory, this pattern should be interpreted with caution. Studies using direct measures of total and regional fat, such as dual-energy X-ray absorptiometry or computed tomography, are needed to clarify the role of adiposity in this association.

Major strengths of this study include its longitudinal design with repeated assessments of salt intake habit scores, VMS, and relevant covariates, as well as the use of both mixed-effects and time-to-event analyses. Inverse probability weighting was applied to account for potential selection bias, and sensitivity analyses using a continuous VMS score, excluding participants with hypertension, and lagging salt intake habit scores by one study visit supported the robustness of the findings. The availability of multiple body composition measures also allowed us to explore potential effect modification by adiposity.

This study has several limitations. First, both salt intake habits and VMS were obtained from self-administered questionnaires rather than from direct measurements. The salt intake habit score summarizes salt-related eating practices, and it should not be read as a measure of total sodium intake. In its development study, the score was only modestly correlated with 24 h urinary sodium excretion (Spearman’s partial r = 0.26) [29], and in the present cohort it could not be compared with urinary sodium or with sodium and potassium intake estimated from a food frequency questionnaire. Some participants were therefore likely to have been placed in an incorrect salt intake category. If such misclassification was unrelated to vasomotor symptom status, it would generally be expected to weaken the observed associations, although bias in either direction is possible for an exposure categorized into tertiles [68]. VMS are inherently subjective and were assessed with the MENQOL, a validated instrument [33,34]. However, because both the exposure and the outcome were self-reported and could not be validated against objective measures in our study population, misclassification of both measures cannot be excluded. Second, because the salt questionnaire was optional, a substantial proportion of participants (n = 1191) were excluded because of missing salt intake habit data, and 2061 of the 3664 included participants (56.3%) had baseline measurements only. These groups differed in some baseline characteristics compared with their respective comparison groups. Although we applied inverse probability weights to account for both the initial exclusion and the availability of repeated measurements, residual selection bias due to unmeasured factors associated with both participation and VMS may remain. Third, salt-related eating behavior is likely to be correlated with other dietary characteristics, such as total energy intake, consumption of processed and spicy foods, caffeine intake, and intake of potassium, magnesium, vegetables, and fruits, some of which may influence VMS. Although we adjusted for socioeconomic and lifestyle factors that tend to cluster with dietary behaviors, information on overall diet quality or nutrient intake was not available. In addition, changes in socioeconomic circumstances during follow-up, such as income and employment, were not fully captured. Therefore, residual confounding by other dietary and social factors cannot be ruled out. Fourth, BMI and hypertension were included as time-varying covariates because they may be related to both salt-related eating behavior and VMS, although they could also partly lie downstream of salt intake. Their inclusion resulted in minimal attenuation of the estimates, indicating that the association was not materially dependent on adjustment for these factors, but this does not exclude mediation. Because hypertension may lead to subsequent salt reduction, reverse causation also remains possible. Findings were similar after excluding participants with hypertension and when salt intake was lagged by one visit; nevertheless, residual time-varying confounding, reverse causation, and mediation through blood pressure or adiposity cannot be ruled out. Fifth, women using hormone replacement therapy at baseline were excluded, but information on nonhormonal treatments for VMS, such as antidepressants and gabapentin, was not available, and their use may have influenced symptom reporting. Sixth, analyses of effect modification by body composition were exploratory and involved multiple comparisons. When we applied Bonferroni correction, the suggestive interaction with percentage body fat did not meet the corrected significance threshold. These findings should therefore be interpreted cautiously and require confirmation in other populations. Seventh, we did not have data on salt sensitivity of blood pressure or on physiological pathways, such as thermoregulatory or vascular responses, that might link salt intake to VMS. Therefore, the mechanisms underlying the observed association could not be examined. Finally, external validity may be limited. Participants were predominantly well-educated Korean women participating in health screening and reported fewer financial difficulties than the general population [19,69]. Moreover, the study was conducted in a setting with relatively high sodium consumption. Replication in populations differing in ethnicity, socioeconomic background, dietary culture, and habitual sodium intake will be important.

## 5. Conclusions

Higher salt intake habit scores were associated with higher odds of bothersome VMS during the menopausal transition. Exploratory analyses did not provide consistent evidence that this association differed by adiposity. Given the observational design, the self-reported exposure and outcome, and the possibility of residual confounding, these findings should be considered hypothesis-generating. Further studies using more objective measures of sodium intake are needed to confirm the association and to clarify whether adiposity influences the relationship between salt intake and VMS.

## Figures and Tables

**Table 1:** Baseline characteristics (n = 3664).

Variable	Frequency (%)
Age at baseline ^1^	45.6 ± 3.1
Menopausal stage	
Premenopause	2829 (77.2)
Early transition	544 (14.8)
Late transition	183 (5.0)
Postmenopause	108 (2.9)
Age at menarche	
<12 years	121 (3.3)
12–13 years	1239 (33.8)
14–16 years	2119 (57.8)
≥17 years	143 (3.9)
Unknown	42 (1.1)
Smoking	
Never	3101 (84.6)
Ever (Current/Former)	397 (10.8)
Unknown	166 (4.5)
Alcohol consumption	
<10 g/day	3083 (84.1)
≥10 g/day	300 (8.2)
Unknown	281 (7.7)
Parity	
Nulliparous	272 (7.4)
Parous	3252 (88.8)
Unknown	140 (3.8)
Marital status	
Married/Cohabitating	3387 (92.4)
Unmarried	148 (4.0)
Divorced/Separated/Widowed	80 (2.2)
Unknown	49 (1.3)
Education	
≤High school	756 (20.6)
≥College	2826 (77.1)
Unknown	82 (2.2)
Body mass index	
<23.0 kg/m^2^	2286 (62.4)
23.0–24.9 kg/m^2^	726 (19.8)
≥25.0 kg/m^2^	652 (17.8)
Waist circumference	
<80 cm	2613 (71.3)
≥80 cm	1051 (28.7)
Percentage body fat	
<35%	2867 (78.2)
≥35%	797 (21.8)
Physical activity ^2^	
Inactivity	1591 (43.4)
Moderate activity	1592 (43.4)
Health-enhancing physical activity	465 (12.7)
Unknown	16 (0.4)
Hypertension ^3^	
Yes	178 (4.9)
Diabetes ^4^	
Yes	82 (2.2)
Medication for hyperlipidemia	
Yes	76 (2.1)

^1^ Baseline age is presented as the mean and standard deviation (SD). ^2^ Physical activity was assessed using the Korean version short form of the International Physical Activity Questionnaire. ^3^ Hypertension was defined as a systolic blood pressure of ≥140 mmHg, a diastolic blood pressure of ≥90 mmHg, history of hypertension or use of antihypertensive drugs. ^4^ Diabetes was defined as glucose ≥126 mg/dL, HbA1c ≥ 6.5%, history of diabetes or use of glucose-lowering medications.

**Table 2:** Associations between salt intake habits and vasomotor symptoms (n = 3664).

Variable	Odds Ratios (95% CI)	
	Model 1	Model 2
Time-varying age (year)	1.14 (1.10–1.17)	1.14 (1.11–1.18)
Salt intake habit scores		
Tertile 1 (≤10)	Ref (1)	Ref (1)
Tertile 2 (11–16)	1.52 (1.25–1.84)	1.48 (1.22–1.80)
Tertile 3 (≥17)	1.55 (1.23–1.94)	1.51 (1.20–1.89)
Menopausal transition		
Premenopause	Ref (1)	Ref (1)
Early transition	1.46 (1.15–1.85)	1.47 (1.16–1.87)
Late transition	3.09 (2.36–4.05)	3.26 (2.48–4.28)
Postmenopause	3.49 (2.57–4.76)	4.10 (2.99–5.62)

Salt intake habit score was the main exposure. Model 1 was adjusted for age and menopausal transition. Model 2 was additionally adjusted for smoking status, parity, marital status, education level, physical activity level, body mass index, hypertension, and age at menarche. All explanatory variables were treated as time-varying, except age at menarche, which was treated as a fixed covariate. Inverse probability weights (the product of weights for baseline inclusion and for repeated measurements) were applied in both models. Mixed-effects models included a participant-specific random intercept.

**Table 3:** Odds ratios for VMS according to tertiles of the salt intake habit score, stratified by body composition (n = 3664).

Variable		Odds Ratios (95% CI)		*p* for Interaction ^1^
	PBF	PBF < 35% n = 2867	PBF ≥ 35% n = 797	
Salt Intake				
Tertile 1 (≤10)		Ref (1) [n = 1131]	Ref (1) [n = 281]	0.032 ^1^
Tertile 2 (11–16)		1.52 (1.22–1.90) [n = 1094]	1.44 (0.99–2.10) [n = 318]	
Tertile 3 (≥17)		1.30 (0.996–1.70) [n = 642]	2.24 (1.46–3.46) [n = 198]	
Tertile 3 vs. Tertile 2 ^2^		0.85 (0.67–1.10); *p* = 0.218 ^2^	1.55 (1.04–2.32); *p* = 0.030 ^2^	
	**WC**	**WC** **< 80 cm** **n = 2613**	**W** **C** **≥ 80 cm** **n = 1051**	
**Salt Intake**				
Tertile 1 (≤10)		Ref (1) [n = 1034]	Ref (1) [n = 378]	0.085 ^1^
Tertile 2 (11–16)		1.40 (1.10–1.77) [n = 1005]	1.71 (1.22–2.38) [n = 407]	
Tertile 3 (≥17)		1.23 (0.93–1.64) [n = 574]	2.08 (1.42–3.04) [n = 266]	
Tertile 3 vs. Tertile 2 ^2^		0.88 (0.68–1.15); *p* = 0.361 ^2^	1.22 (0.86–1.72); *p* = 0.266 ^2^	
	**BMI**	**BMI** **< 23 kg/m** **^2^** **n = 2286**	**BMI** **≥ 23 kg/m** **^2^** **n = 1378**	
**Salt Intake**				
Tertile 1 (≤10)		Ref (1) [n = 902]	Ref (1) [n = 510]	0.289 ^1^
Tertile 2 (11–16)		1.50 (1.16–1.93) [n = 865]	1.49 (1.11–2.00) [n = 547]	
Tertile 3 (≥17)		1.33 (0.99–1.79) [n = 519]	1.81 (1.28–2.56) [n = 321]	
Tertile 3 vs. Tertile 2 ^2^		0.89 (0.67–1.17); *p* = 0.400 ^2^	1.22 (0.88–1.68); *p* = 0.230 ^2^	

Each body composition measure was examined in a separate model by adding its product term with salt intake habit tertile. Models accounted for age, menopausal stage, smoking, parity, marital status, education, and physical activity using updated values over follow-up, whereas age at menarche was retained at baseline. Participant-level random intercepts and inverse probability weights (the product of weights for baseline inclusion and for repeated measurements) were applied. Although salt intake habits and body composition were treated as time-varying, values in brackets indicate the number of observed participants in each cell at baseline to show the initial distribution. ^1^ Overall evidence for interaction was evaluated with a Wald test. Statistical significance was defined as *p* < 0.0167 after Bonferroni correction for three body composition measures (0.05/3). ^2^ Odds ratio for tertile 3 relative to tertile 2 within each stratum, estimated from the same model using a Wald test. Statistical significance was defined as *p* < 0.0083 after Bonferroni correction for six comparisons (three body composition measures × two strata; 0.05/6).

## Data Availability

The data supporting the findings of this study are not publicly available at present, but the analytical methods and dataset are available from the corresponding author upon request.

## Supplementary Materials

The following supporting information can be downloaded at: https://www.mdpi.com/article/10.3390/nu18213618/s1, Figure S1. Directed acyclic graph of the hypothesized relationships between salt intake habits and bothersome vasomotor symptoms; Figure S2. Distribution of the MENQOL vasomotor domain score at baseline (n = 3664). Bars indicate the proportion of participants in each category of the MENQOL vasomotor domain score (range 1–8). A score of 1 indicates no symptoms, and a score >1 and ≤2 indicates symptoms that were present but not bothersome. Bothersome VMS, the primary outcome, was defined as a score >2. MENQOL = Menopause-Specific Quality of Life questionnaire; VMS = vasomotor symptoms; Figure S3. Predicted prevalence of VMS according to tertiles of the salt intake habit score, stratified by body composition (n = 3664). Marginal predicted probabilities of bothersome VMS according to tertiles of the salt intake habit score, stratified by (A) percentage body fat (<35% vs. ≥35%), (B) waist circumference (<80 vs. ≥80 cm), and (C) body mass index (<23 vs. ≥23kg/m^2^). Error bars indicate 95% confidence intervals. *p*-values for interaction were calculated using Wald tests for the interaction terms between tertiles of the salt intake habit score and each body composition measure; each model was fitted once, and all subsequent estimates were obtained from the fitted model using postestimation; statistical significance was defined as *p* < 0.0167 after Bonferroni correction for three body composition measures. BMI = body mass index; PBF = percentage body fat; VMS = vasomotor symptoms; WC = waist circumference; Figure S4. Predicted prevalence of VMS according to tertiles of the salt intake habit score, stratified by BMI with a cut-off of 25 kg/m^2^ (<25 vs. ≥25 kg/m^2^) (n = 3664). Marginal predicted probabilities of bothersome VMS according to tertiles of the salt intake habit score, stratified by body mass index using 25 kg/m^2^ as the cut-off. Error bars indicate 95% confidence intervals. The *p*-value for interaction was calculated using a Wald test for the interaction term between tertiles of the salt intake habit score and body mass index category. BMI = body mass index; VMS = vasomotor symptoms; Figure S5. Cumulative incidence of VMS by tertiles of the salt intake habit score (n = 1312). Kaplan–Meier curves show the cumulative incidence of bothersome VMS according to baseline tertiles of the salt intake habit score: tertile 1 (≤10), tertile 2 (11–16), and tertile 3 (≥17). Differences across tertiles were assessed using the log-rank test. Numbers at risk are shown below the plot. VMS = vasomotor symptoms. Table S1: Baseline characteristics according to inclusion in the analytic sample. Table S2. Baseline characteristics according to follow-up measurement status. Table S3. Associations between vasomotor symptoms and covariates (n = 3664). Table S4. Associations between salt intake habits and the MENQOL vasomotor domain score as a continuous outcome (n = 3664). Table S5. Associations between salt intake habits and vasomotor symptoms after excluding individuals with hypertension at any study visit (n = 3377). Table S6. Associations between salt intake habits and vasomotor symptoms after additional adjustment for alcohol consumption (n = 3664). Table S7. Associations between salt intake habits and vasomotor symptoms using exposure lagged by one study visit (n = 1603). Table S8. Hazard ratios for incident vasomotor symptoms by baseline and time-varying salt intake habits (n = 1312).
