# Supplementary material for: Association Between Salt Intake Habits and Vasomotor Symptoms According to Adiposity Among Middle-Aged Korean Women: A Longitudinal Cohort Study
Source: Nutrients. 2026 Oct 31;18(21):3618. doi: 10.3390/nu18213618 (PMC13765249; doi:10.3390/nu18213618)
Supplement: Supplementary file 1 [file nutrients-18-03618-s001.zip › Supplementary_Tables_R/Table S1.pdf]

Table S1. Baseline characteristics according to inclusion in the analytic sample

Variable	Excluded (n = 1,352)	Included (n = 3,664)	P value	SMD
Age at baseline[1]	44.6 ± 2.3	45.6 ± 3.1	<0.001	0.354
Menopausal stage			<0.001	
Premenopause	1,072 (79.3)	2,829 (77.2)		0.050
Early transition	218 (16.1)	544 (14.9)		0.035
Late transition	43 (3.2)	183 (5.0)		0.092
Postmenopause	8 (0.6)	108 (3.0)		0.179
Unknown	11 (0.8)	0 (0.0)		0.128
Age at menarche			0.115	
<12 years	50 (3.7)	121 (3.3)		0.022
12–13 years	489 (36.2)	1,239 (33.8)		0.049
14–16 years	733 (54.2)	2,119 (57.8)		0.073
≥17 years	56 (4.1)	143 (3.9)		0.012
Unknown	24 (1.8)	42 (1.1)		0.052
Smoking			<0.001	
Never	1,126 (83.3)	3,101 (84.6)		0.037
Ever (Current/Former)	191 (14.1)	397 (10.8)		0.100
Unknown	35 (2.6)	166 (4.5)		0.105
Alcohol consumption			0.006	
<10 g/day	1,162 (86.0)	3,083 (84.1)		0.051
≥10 g/day	121 (9.0)	300 (8.2)		0.027
Unknown	69 (5.1)	281 (7.7)		0.105
Parity			0.006	
Nulliparous	106 (7.8)	272 (7.4)		0.016
Parous	1,167 (86.3)	3,252 (88.8)		0.074
Unknown	79 (5.8)	140 (3.8)		0.094
Marital status			<0.001	
Married/Cohabitating	1,198 (88.6)	3,387 (92.4)		0.131
Unmarried	72 (5.3)	148 (4.0)		0.061
Divorced/Separated/Widowed	23 (1.7)	80 (2.2)		0.035
Unknown	59 (4.4)	49 (1.3)		0.183
Education			<0.001	
≤High school	208 (15.4)	756 (20.6)		0.137
≥College	1,072 (79.3)	2,826 (77.1)		0.052
Unknown	72 (5.3)	82 (2.2)		0.162
Body mass index			0.003	
<23.0 kg/m^2	910 (67.3)	2,286 (62.4)		0.103
23.0–24.9 kg/m^2	243 (18.0)	726 (19.8)		0.047
≥25.0 kg/m^2	198 (14.6)	652 (17.8)		0.086
Unknown	1 (0.1)	0 (0.0)		0.038
Waist circumference			0.007	
<80 cm	987 (73.0)	2,613 (71.3)		0.038
≥80 cm	362 (26.8)	1,051 (28.7)		0.043
Unknown	3 (0.2)	0 (0.0)		0.067
Percentage body fat			0.063	
<35%	1,089 (80.7)	2,867 (78.3)		0.057
≥35%	261 (19.3)	797 (21.8)		0.061
Unknown	2 (0.1)	0 (0.0)		0.054
Physical activity[2]			<0.001	
Inactivity	701 (51.9)	1,591 (43.4)		0.169
Moderate activity	512 (37.9)	1,592 (43.5)		0.114
Health-enhancing physical activity	117 (8.7)	465 (12.7)		0.131
Unknown	22 (1.6)	16 (0.4)		0.118
Hypertension[3]			0.216	

No	1,300 (96.2)	3,484 (95.1)		0.052
Yes	52 (3.8)	178 (4.9)		0.050
Unknown	0 (0.0)	2 (0.1)		0.033
Diabetes [4]			0.091	
No	1,332 (98.5)	3,582 (97.8)		0.056
Yes	20 (1.5)	82 (2.2)		0.056
Medication for hyperlipidemia			0.085	
No	1,334 (98.7)	3,588 (97.9)		0.057
Yes	18 (1.3)	76 (2.1)		0.057

Data on participants who withdrew from the study (n = 230) were unavailable for comparison because, according to the study protocol, all of their data were deleted upon withdrawal.

P values were obtained using the t-test for continuous variables and the chi-square test for categorical variables. For continuous variables, the standardized mean difference (SMD) was calculated as the difference in means divided by the pooled standard deviation; for categorical variables, SMDs were calculated for each category based on the proportion of participants in that category. An absolute SMD > 0.1 was considered indicative of a meaningful imbalance.

[1] Baseline age is presented as the mean and standard deviation (SD). [2] Physical activity was assessed using the Korean version short form of the International Physical Activity Questionnaire. [3] Hypertension was defined as a systolic blood pressure of ≥140 mmHg, a diastolic blood pressure of ≥90 mmHg, history of hypertension or use of antihypertensive drugs. [4] Diabetes was defined as glucose ≥126 mg/dL, HbA1c ≥6.5, history of diabetes or use of glucose-lowering medications.
