# Supplementary material for: Association Between Salt Intake Habits and Vasomotor Symptoms According to Adiposity Among Middle-Aged Korean Women: A Longitudinal Cohort Study
Source: Nutrients. 2026 Oct 31;18(21):3618. doi: 10.3390/nu18213618 (PMC13765249; doi:10.3390/nu18213618)
Supplement: Supplementary file 1 [file nutrients-18-03618-s001.zip › Supplementary_Tables_R/Table S2.pdf]

Table S2. Baseline characteristics according to follow-up measurement status

Variable	Baseline only (n = 2,061)	Repeated measurements (n = 1,603)	P value	SMD
Age at baseline [1]	45.7 ± 3.2	45.5 ± 2.9	0.103	0.055
Menopausal stage			<0.001	
Premenopause	1,592 (77.2)	1,237 (77.2)		0.002
Early transition	289 (14.0)	255 (15.9)		0.053
Late transition	100 (4.9)	83 (5.2)		0.015
Postmenopause	80 (3.9)	28 (1.8)		0.129
Age at menarche			<0.001	
<12 years	71 (3.4)	50 (3.1)		0.018
12–13 years	715 (34.7)	524 (32.7)		0.042
14–16 years	1,156 (56.1)	963 (60.1)		0.081
≥17 years	80 (3.9)	63 (3.9)		0.003
Unknown	39 (1.9)	3 (0.2)		0.169
Smoking			0.001	
Never	1,740 (84.4)	1,361 (84.9)		0.013
Ever (Current/Former)	207 (10.0)	190 (11.9)		0.058
Unknown	114 (5.5)	52 (3.2)		0.112
Alcohol consumption			0.001	
<10 g/day	1,694 (82.2)	1,389 (86.7)		0.123
≥10 g/day	190 (9.2)	110 (6.9)		0.087
Unknown	177 (8.6)	104 (6.5)		0.080
Parity			0.019	
Nulliparous	152 (7.4)	120 (7.5)		0.004
Parous	1,814 (88.0)	1,438 (89.7)		0.054
Unknown	95 (4.6)	45 (2.8)		0.095
Marital status			<0.001	
Married/Cohabitating	1,885 (91.5)	1,502 (93.7)		0.085
Unmarried	80 (3.9)	68 (4.2)		0.018
Divorced/Separated/Widowed	55 (2.7)	25 (1.6)		0.077
Unknown	41 (2.0)	8 (0.5)		0.135
Education			0.003	
≤High school	431 (20.9)	325 (20.3)		0.016
≥College	1,569 (76.1)	1,257 (78.4)		0.055
Unknown	61 (3.0)	21 (1.3)		0.114
Body mass index			0.283	
<23.0 kg/m2	1,272 (61.7)	1,014 (63.3)		0.032
23.0–24.9 kg/m2	404 (19.6)	322 (20.1)		0.012
≥25.0 kg/m2	385 (18.7)	267 (16.7)		0.053
Waist circumference			0.125	
<80 cm	1,449 (70.3)	1,164 (72.6)		0.051
≥80 cm	612 (29.7)	439 (27.4)		0.051
Percentage body fat			0.112	
<35%	1,593 (77.3)	1,274 (79.5)		0.053
≥35%	468 (22.7)	329 (20.5)		0.053
Physical activity [2]			0.064	
Inactivity	915 (44.4)	676 (42.2)		0.045
Moderate activity	859 (41.7)	733 (45.7)		0.082
Health-enhancing physical activity	276 (13.4)	189 (11.8)		0.048
Unknown	11 (0.5)	5 (0.3)		0.034
Hypertension [3]			0.141	
No	1,949 (94.6)	1,535 (95.8)		0.056
Yes	110 (5.3)	68 (4.2)		0.051
Unknown	2 (0.1)	0 (0.0)		0.044
Diabetes [4]			0.026	

No	2,005 (97.3)	1,577 (98.4)		0.075
Yes	56 (2.7)	26 (1.6)		0.075
Medication for hyperlipidemia			0.321	
No	2,014 (97.7)	1,574 (98.2)		0.033
Yes	47 (2.3)	29 (1.8)		0.033

Baseline only indicates participants with a single analytic measurement at baseline. Repeated measurements indicates participants with at least one additional assessment after baseline.

P values were obtained using the t-test for continuous variables and the chi-square test for categorical variables. For continuous variables, the standardized mean difference (SMD) was calculated as the difference in means divided by the pooled standard deviation; for categorical variables, SMDs were calculated for each category based on the proportion of participants in that category. An absolute SMD > 0.1 was considered indicative of a meaningful imbalance.

[1] Baseline age is presented as the mean and standard deviation (SD). [2] Physical activity was assessed using the Korean version short form of the International Physical Activity Questionnaire. [3] Hypertension was defined as a systolic blood pressure of ≥140 mmHg, a diastolic blood pressure of ≥90 mmHg, history of hypertension or use of antihypertensive drugs. [4] Diabetes was defined as glucose ≥126 mg/dL, HbA1c ≥6.5, history of diabetes or use of glucose-lowering medications.
