# Supplementary material for: Association Between Salt Intake Habits and Vasomotor Symptoms According to Adiposity Among Middle-Aged Korean Women: A Longitudinal Cohort Study
Source: Nutrients. 2026 Oct 31;18(21):3618. doi: 10.3390/nu18213618 (PMC13765249; doi:10.3390/nu18213618)
Supplement: Supplementary file 1 [file nutrients-18-03618-s001.zip › Supplementary_Tables_R/Table S3.pdf]

Table S3. Associations between vasomotor symptoms and covariates (n = 3,664)

Variable	OR (95% CI)	P value
Age at menarche		
<12 years old	Ref (1)	
12-13 years old	0.76 (0.43-1.34)	0.346
14–16 years old	0.86 (0.49-1.49)	0.586
≥17 years old	0.74 (0.35-1.54)	0.419
Smoking		
Never	Ref (1)	
Ever (Current/Former)	1.21 (0.89-1.64)	0.221
Body mass index		
<23.0 kg/m^2	Ref (1)	
23.0–24.9 kg/m^2	1.46 (1.17-1.82)	<0.001
≥25.0 kg/m^2	2.49 (1.95-3.19)	<0.001
Physical activity		
Inactive	Ref (1)	
Moderately active	0.94 (0.79-1.13)	0.538
Health enhancing physical activity	0.78 (0.60-1.01)	0.056
Parity		
Nulliparous	Ref (1)	
Parous	0.69 (0.44-1.09)	0.113
Marital status		
Married/cohabitating	Ref (1)	
Unmarried	0.90 (0.48-1.67)	0.733
Divorced/separated/widowed	1.71 (0.91-3.21)	0.097
Education		
≤High school	Ref (1)	
≥College	0.94 (0.74-1.20)	0.637
Hypertension		
No	Ref (1)	
Yes	1.69 (1.20-2.37)	0.002

All covariates are time-variant, except for age at menarche. Odds ratios were obtained from Model 2 of Table 2, in which salt intake habit score and all listed covariates were included simultaneously, with participant-level random intercepts and inverse probability weights (the product of weights for baseline inclusion and for repeated measurements).
