# Supplementary material for: Association Between Salt Intake Habits and Vasomotor Symptoms According to Adiposity Among Middle-Aged Korean Women: A Longitudinal Cohort Study
Source: Nutrients. 2026 Oct 31;18(21):3618. doi: 10.3390/nu18213618 (PMC13765249; doi:10.3390/nu18213618)
Supplement: Supplementary file 1 [file nutrients-18-03618-s001.zip › Supplementary_Tables_R/Table S4.pdf]

Table S4. Associations between salt intake habits and the MENQOL vasomotor domain score as a continuous outcome (n = 3,664)

Variable	β (95% CI)	
	Model 1	Model 2
Time-varying age (year)	0.05 (0.04-0.06)	0.05 (0.04-0.06)
Salt intake habit scores		
Tertile 1 (≤10)	Ref (0)	Ref (0)
Tertile 2 (11–16)	0.09 (0.03-0.16)	0.08 (0.02-0.15)
Tertile 3 (≥17)	0.17 (0.09-0.24)	0.15 (0.07-0.23)
Menopausal transition		
Premenopause	Ref (0)	Ref (0)
Early transition	0.14 (0.06-0.22)	0.13 (0.05-0.22)
Late transition	0.55 (0.46-0.65)	0.56 (0.47-0.66)
Postmenopause	0.64 (0.53-0.75)	0.69 (0.58-0.80)

The MENQOL vasomotor domain score (range 1–8) was analyzed as a continuous outcome using linear mixed-effects models with a participant-specific random intercept. Salt intake habit score was the main exposure. Model 1 was adjusted for age and menopausal transition. Model 2 was additionally adjusted for smoking status, parity, marital status, education level, physical activity level, body mass index, hypertension, and age at menarche. All explanatory variables were treated as time-varying, except age at menarche, which was treated as a fixed covariate. Inverse probability weights (the product of weights for baseline inclusion and for repeated measurements) were applied in both models.
