# Supplementary material for: Association Between Salt Intake Habits and Vasomotor Symptoms According to Adiposity Among Middle-Aged Korean Women: A Longitudinal Cohort Study
Source: Nutrients. 2026 Oct 31;18(21):3618. doi: 10.3390/nu18213618 (PMC13765249; doi:10.3390/nu18213618)
Supplement: Supplementary file 1 [file nutrients-18-03618-s001.zip › Supplementary_Tables_R/Table S5.pdf]

Table S5. Associations between salt intake habits and vasomotor symptoms after excluding individuals with hypertension at any study visit (n = 3,377)

Variable	VMS (binary)		VMS score (continuous)	
	OR (95% CI)	P value	β (95% CI)	P value
Salt intake habit scores				
Tertile 1 (≤10)	Ref (1)		Ref (0)	
Tertile 2 (11–16)	1.52 (1.24-1.87)	<0.001	0.09 (0.03-0.16)	0.006
Tertile 3 (≥17)	1.55 (1.21-1.97)	<0.001	0.15 (0.07-0.23)	<0.001

Odds ratios and β coefficients were obtained using the same specification as Model 2 of Table 2, excluding hypertension as a covariate. Vasomotor symptoms were analyzed as a binary outcome (score > 2 vs. ≤ 2; scores ≤ 2 indicate no symptoms or symptoms that were present but not bothersome) using mixed-effects logistic regression, and the MENQOL vasomotor domain score (range 1–8) was analyzed as a continuous outcome using linear mixed-effects models. The models were adjusted for age, menopausal stage, smoking status, parity, marital status, education level, physical activity level, and body mass index as time-varying covariates, and for age at menarche as a fixed covariate, with participant-level random intercepts and inverse probability weights (the product of weights for baseline inclusion and for repeated measurements).
