# Supplementary material for: Association Between Salt Intake Habits and Vasomotor Symptoms According to Adiposity Among Middle-Aged Korean Women: A Longitudinal Cohort Study
Source: Nutrients. 2026 Oct 31;18(21):3618. doi: 10.3390/nu18213618 (PMC13765249; doi:10.3390/nu18213618)
Supplement: Supplementary file 1 [file nutrients-18-03618-s001.zip › Supplementary_Tables_R/Table S7.pdf]

Table S7. Associations between salt intake habits and vasomotor symptoms using exposure lagged by one study visit (n = 1,603)

Variable	VMS (binary)		VMS score (continuous)	
	OR (95% CI)	P value	β (95% CI)	P value
Salt intake habit scores				
Tertile 1 (≤10)	Ref (1)		Ref (0)	
Tertile 2 (11–16)	1.26 (0.95-1.66)	0.108	0.12 (0.01-0.22)	0.034
Tertile 3 (≥17)	1.64 (1.18-2.28)	0.003	0.23 (0.10-0.36)	0.001

Odds ratios and β coefficients were obtained using the same specification as Model 2 of Table 2, except that salt intake habits were lagged by one study visit (i.e., salt intake habits at visit t and VMS at visit t + 1) to reduce potential reverse causation. Covariates were not lagged. Vasomotor symptoms were analyzed as a binary outcome (score > 2 vs. ≤ 2; scores ≤ 2 indicate no symptoms or symptoms that were present but not bothersome) using mixed-effects logistic regression, and the MENQOL vasomotor domain score (range 1–8) was analyzed as a continuous outcome using linear mixed-effects models. The models were adjusted for age, menopausal stage, smoking status, parity, marital status, education level, physical activity level, hypertension, and body mass index as time-varying covariates, and for age at menarche as a fixed covariate, with participant-level random intercepts and inverse probability weights (the product of weights for baseline inclusion and for repeated measurements).
