# Supplementary material for: Association Between Salt Intake Habits and Vasomotor Symptoms According to Adiposity Among Middle-Aged Korean Women: A Longitudinal Cohort Study
Source: Nutrients. 2026 Oct 31;18(21):3618. doi: 10.3390/nu18213618 (PMC13765249; doi:10.3390/nu18213618)
Supplement: Supplementary file 1 [file nutrients-18-03618-s001.zip › Supplementary_Tables_R/Table S8.pdf]

Table S8. Hazard ratios for incident vasomotor symptoms by baseline and time-varying salt intake habits (n = 1,312)

Variable	Person year (PY)	Incident cases	Incidence density (/1000 PY)	Age-adjusted HR (95% CI)	Multivariable-adjusted HR (95% CI)	HR (95% CI) in the model with time-dependent variables
Salt intake habit scores						
Tertile 1 (≤10)	2,702.7	190	70.3	Ref (1)	Ref (1)	Ref (1)
Tertile 2 (11–16)	2,364.9	197	83.3	1.14 (0.93-1.40)	1.16 (0.94-1.43)	1.26 (1.02-1.54)
Tertile 3 (≥17)	1,396.1	125	89.5	1.26 (0.995-1.58)	1.25 (0.99-1.60)	1.23 (0.97-1.56)
P trend				0.048	0.052	0.053

Multivariable-adjusted HRs and HRs in the model with time-dependent variables were adjusted for the same covariates as in Model 2 of Table 2. Incident bothersome VMS was analyzed using Cox proportional hazards models, with salt intake habits and covariates treated either as baseline measures or as time-varying variables. In the time-dependent models, values measured at each visit were applied to the subsequent risk interval and used to predict bothersome VMS identified at the next assessment. Inverse probability weights were applied to account for selection into the Cox analytic sample.
